# Supplementary material for: EWSR1-ATF1 dependent 3D connectivity regulates oncogenic and differentiation programs in Clear Cell Sarcoma
Source: Nat Commun. 2022 Apr 27;13:2267. doi: 10.1038/s41467-022-29910-4 (PMC9046276; doi:10.1038/s41467-022-29910-4)
Supplement: Supplementary file 1 — Supplementary Information [file 41467_2022_29910_MOESM1_ESM.pdf]

EWSR1-ATF1 dependent 3D connectivity regulates oncogenic and differentiation programs in Clear Cell Sarcoma

Möller *et al.*

Supplementary Fig. 1

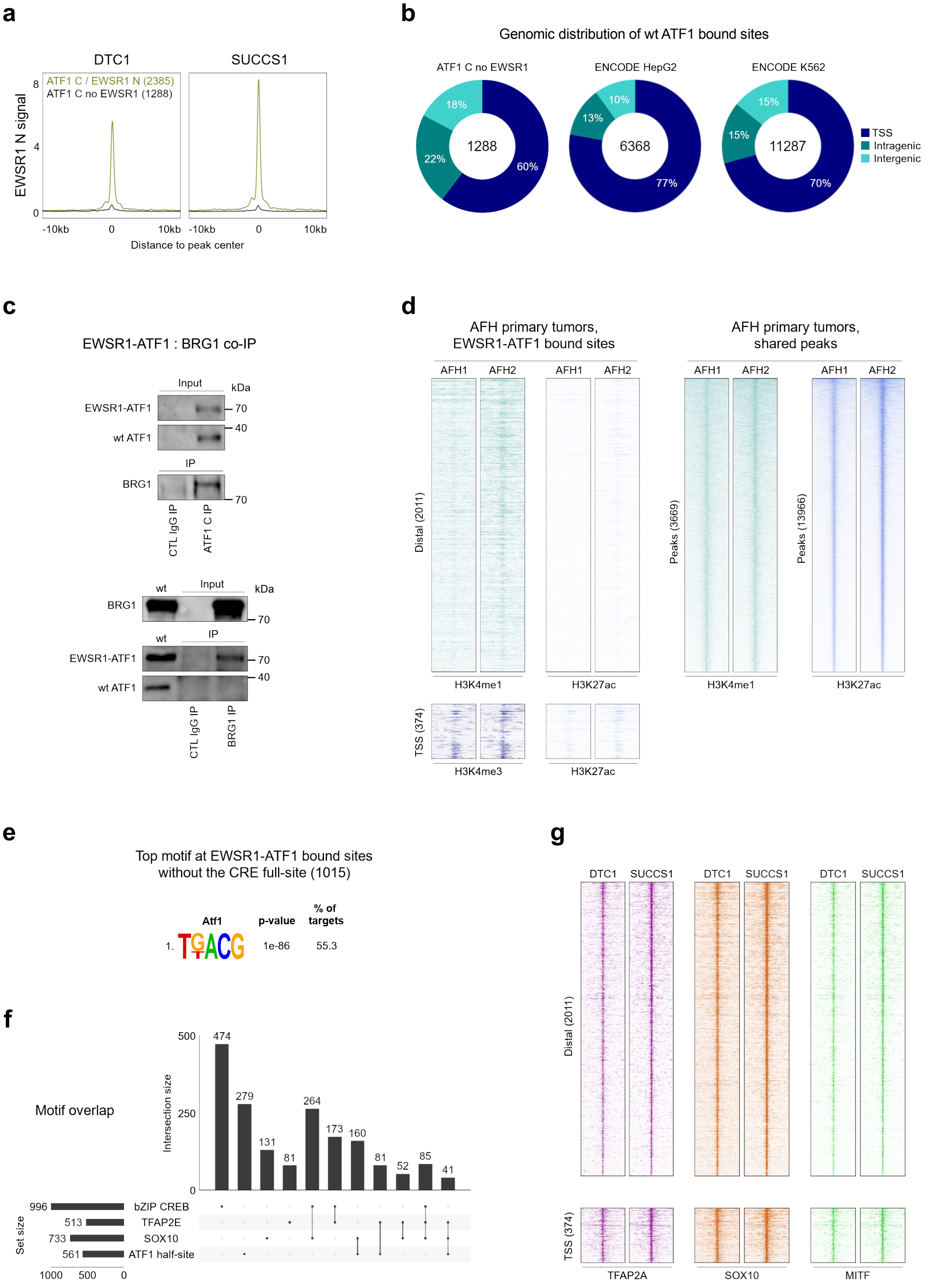

Supplementary Fig. 1: EWSR1-ATF1 binding sites are preferentially activated in CCS tumor cells and co-occupied by NCSC-related TFs.

a, Composite plots showing EWSR1 N ChIP-seq signal at 2385 EWSR1-ATF1 consensus (green line) or 1288 wt ATF1 (black line) binding sites in DTC1 and SU-CCS-1 cell lines. **b,** Genomic distribution of 1288 wt ATF1 sites (*left*) showing their preferential association with TSSs, similar to the binding pattern identified for wt ATF1 in HepG2 and K562 cells (*middle and right*). **c,** Co-IP assay showing that EWSR1-ATF1 is directly and selectively interacting with BRG1 in DTC1 cells. The IP was performed using either anti-ATF1 C (*top*) or anti-BRG1 (*bottom*) antibodies, compared to control (CTL) IgG, and the western blot revealed using the corresponding antibodies (anti-ATF1 C antibody for both EWSR1-ATF1 and wt ATF1). The experiment was independently repeated twice with similar results. Source images are provided in Source Data file. **d,** *Left panel*: Heatmaps depicting H3K4me1-K27ac and H3K4me3-K27ac ChIP-seq signal intensities at 2011 distal (*top*) and 374 proximal (*bottom*) EWSR1-ATF1 binding sites in primary AFH tumors. *Right panel:* Heatmaps showing signal intensities at all H3K4me1 (n=3669) and H3K27ac (n=13966) peaks in common between two primary AFH tumors. For each heatmap 20 kb regions centered on the EWSR1-ATF1 peaks (*left panel*), or on H3K4me1 / H3K27ac peaks (*right panel*) are shown. **e,** *De novo* motif enrichment analysis showing CRE half-site as top motif at 1015 EWSR1-ATF1 distal binding regions lacking full CRE-sites. Binomial p-values are given by the motif enrichment software HOMER. **f,** Upset plot of motifs overlap at 2011 EWSR1-ATF1 distal sites showing the co-enrichment of CRE full- or half sites with TFAP2 and/or SOX motifs. **g,** Heatmaps depicting TFAP2A, SOX10 and MITF signal intensities at 2011 distal (*top*) and 374 TSS-associated (*bottom*) EWSR1-ATF1 sites in DTC1 and SU-CCS-1 cell lines. 20 kb regions centered on EWSR1-ATF1 peaks are shown.

Supplementary Fig. 2

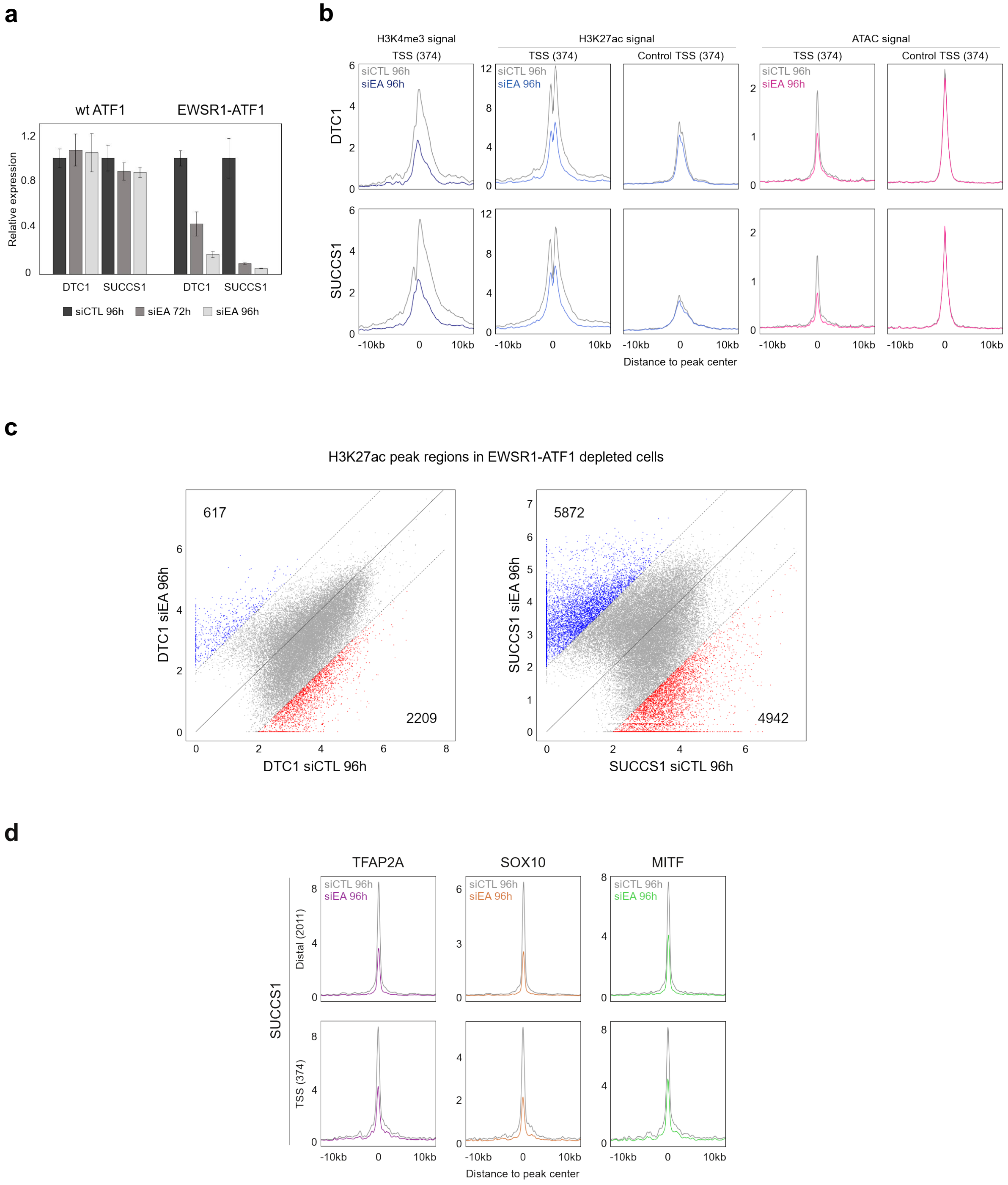

Supplementary Fig. 2: EWSR1-ATF1 depletion leads to H3K27ac and ATAC signals reduction at its binding sites, and the displacement of NCSC-related TFs.

a, qPCR analysis showing reduction of *EWSR1-ATF1* but not wt *ATF1* transcripts in DTC1 and SU-CCS-1 cells treated with an *EWSR1-ATF1* targeting (siEA) or control (siCTL) siRNAs at 72h and/or 96h. Three replicates of each condition were used to calculate mean Ct values, mean relative expression values were then calculated according to the $2^{-\Delta\Delta Ct}$ method relative to the siCTL sample values, and normalized to the endogenous control gene *GAPDH*. Error bars show standard deviation of mean values (n=3 sample replicates). The experiment was independently repeated twice with similar results. Source data are provided in the Source Data file. **b,** Composite plots showing decreases in H3K4me3, H3K27ac and ATAC signals at 374 TSS-associated EWSR1-ATF1 sites in DTC1 and SU-CCS- 1 cells transfected with siEA for 96h, compared to siCTL. No significant changes were observed at 374 active control TSSs not bound by EWSR1-ATF1. **c,** Scatter plots of H3K27ac peak scores in siEA- and siCTL-treated DTC1 and SU-CCS-1 cells. At a FC 2 cutoff, 617 and 5872 peaks were increased in siEA-treated DTC1 and SU-CCS-1, respectively (blue); whereas 2209 and 4942, respectively, were decreased (red). **d,** Composite plots showing reduction in TFAP2A, SOX10 and MITF signals at 2011 Distal- and 374 TSS-associated EWSR1-ATF1 binding sites in siEA-treated SU-CCS-1 cells, as compared to siCTL.

Supplementary Fig. 3

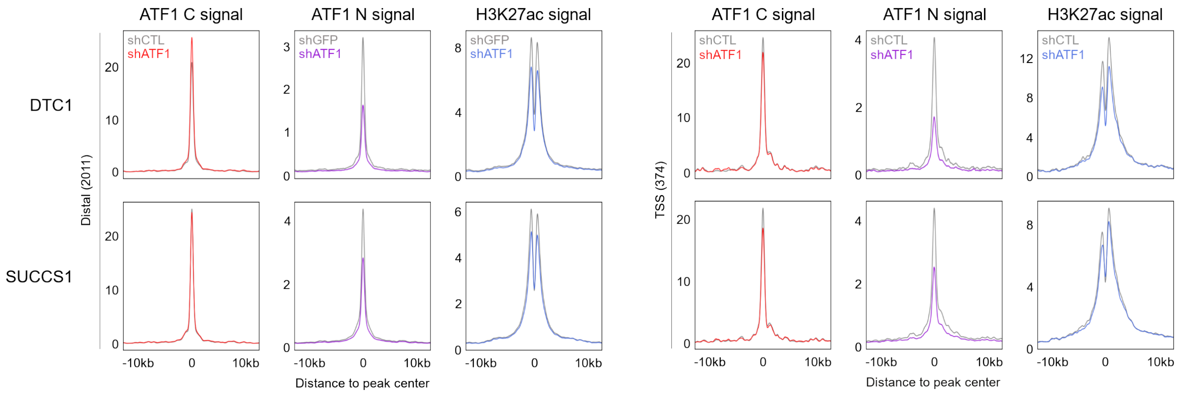

Supplementary Fig. 3: wt ATF1-depletion leads to H3K27ac signal reduction at EWSR1-ATF1 binding sites.

Composite plots showing decreases in ATF1 N and H3K27ac, but not ATF1 C, signals at 2011 distal- and 374 TSS-associated EWSR1-ATF1 sites in DTC1 (*top*) and SU-CCS-1 (*bottom*) cells infected with a shRNA targeting wt ATF1 (shATF1) for 72h, as compared to control (shCTL).

Supplementary Fig. 4

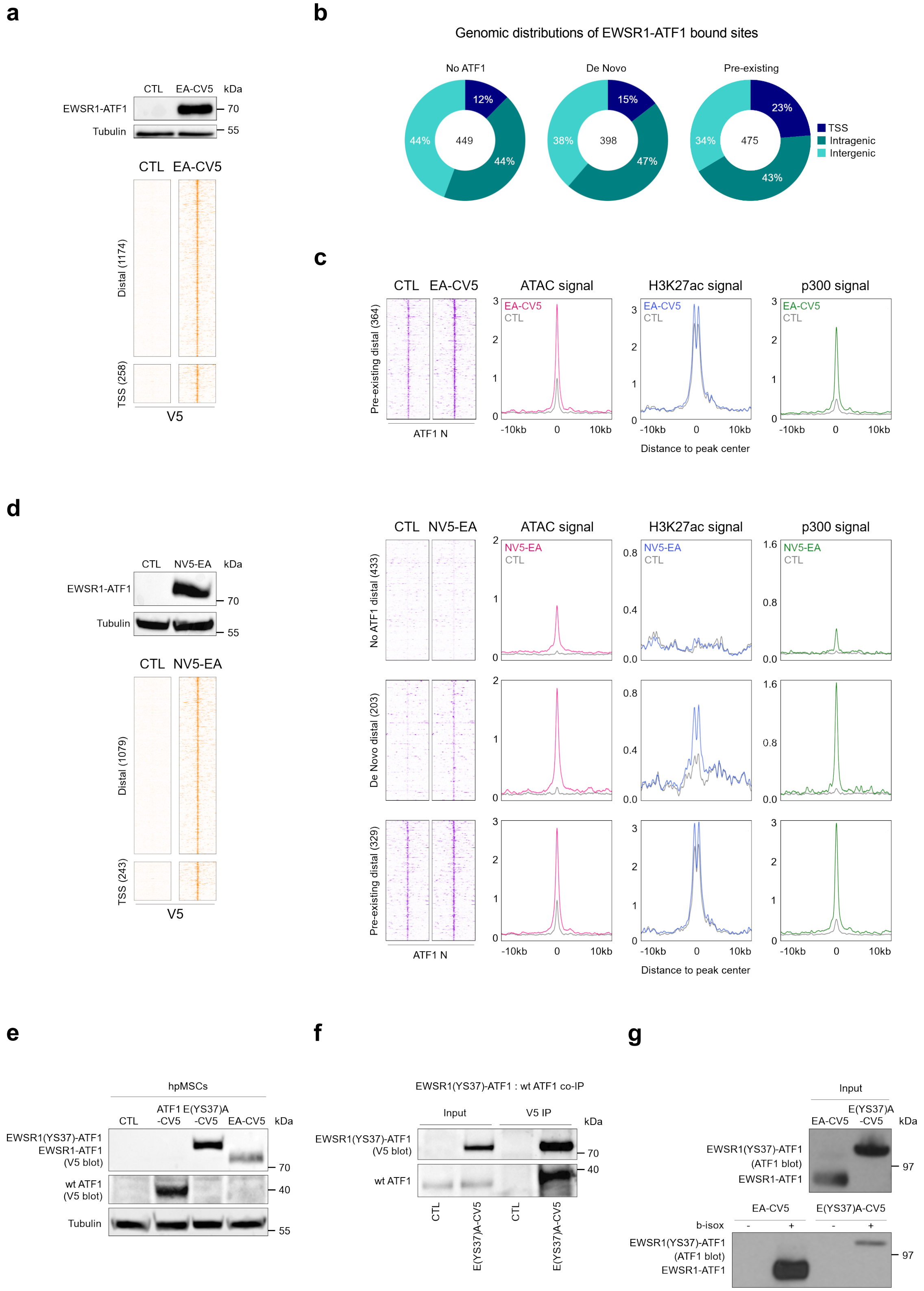

Supplementary Fig. 4: EWSR1-ATF1-mediated chromatin activation in primary human MSCs is dependent on initial wt ATF1 binding pattern.

a, *Top:* Western blot analysis showing the induction of C-terminal V5-tagged EWSR1-ATF1 protein (EA-CV5), compared to control (CTL) in hpMSCs. The fusion protein was detected using an anti-V5 tag antibody. Tubulin signal are shown as loading control. *Bottom:* Heatmap depicting V5 ChIP-seq signal at distal (n=1174) and proximal (n=258) sites shared between CCS cells and EA-CV5-infected hpMSCs. 20 kb regions centered on V5 signal are shown. **b,** Genomic distribution of the different EWSR1-ATF1-bound sites categories *No ATF1* (n=449), *De Novo* (n=398) and *Pre-existing* (n=475) in hpMSCs, showing their preferential distal location. **c,** Composite plots depicting ATAC, H3K27ac and p300 signal intensities at Pre-existing distal sites, showing increases in DNA accessibility and p300 recruitment in EA-CV5 hpMSCs cells. **d,** *Left:* Western blot analysis (*top*) showing the induction of N-terminal V5-tagged EWSR1-ATF1 protein (NV5-EA) in hpMSCs, and V5 ChIP-seq signal heatmap (*bottom*) of the bound sites (1079 distal and 243 proximal) shared with CCS cells, analogous to (**a**). *Right:* Composite plots depicting ATAC, H3K27ac and p300 signal intensities at *No ATF1*, *De Novo* and *Pre-existing* distal sites showing similarities in DNA accessibility and chromatin activation changes between NV5-EA- and EA-CV5-expressing hpMSCs cells. **e,** Western blot analysis showing the induction of C-terminally V5-tagged ATF1 (ATF1-CV5), EWSR1(YS37mutant)-ATF1 (E(YS37)A-CV5) and EA-CV5 proteins in hpMSCs, detected using an anti-V5 tag antibody. Tubulin signals are shown as loading control. **f,** Co-immunoprecipitation (co-IP) assay showing the direct interaction between the EWSR1(YS37mutant)-ATF1 and wt ATF1 proteins in 293T cells transfected with either the E(YS37)A-CV5 or empty vector (CTL) constructs. The IP was performed using an anti-V5 tag antibody, and the western blot revealed using anti-V5 (*top*) or anti- ATF1 C (*bottom*) antibodies. **g,** B-isox precipitation assay in 293T cells after their transfection with either EA-CV5 or E(YS37)A-CV5 construct, showing that EWSR1(YS37mutant)-ATF1 has lost the precipitation properties displayed by EA-CV5. The Western blot signal was revealed using an anti-ATF1 C antibody. All protein over expression and IP experiments in hpMSCs were independently repeated twice with similar results. Source images are provided in the Source Data file.

Supplementary Fig. 5

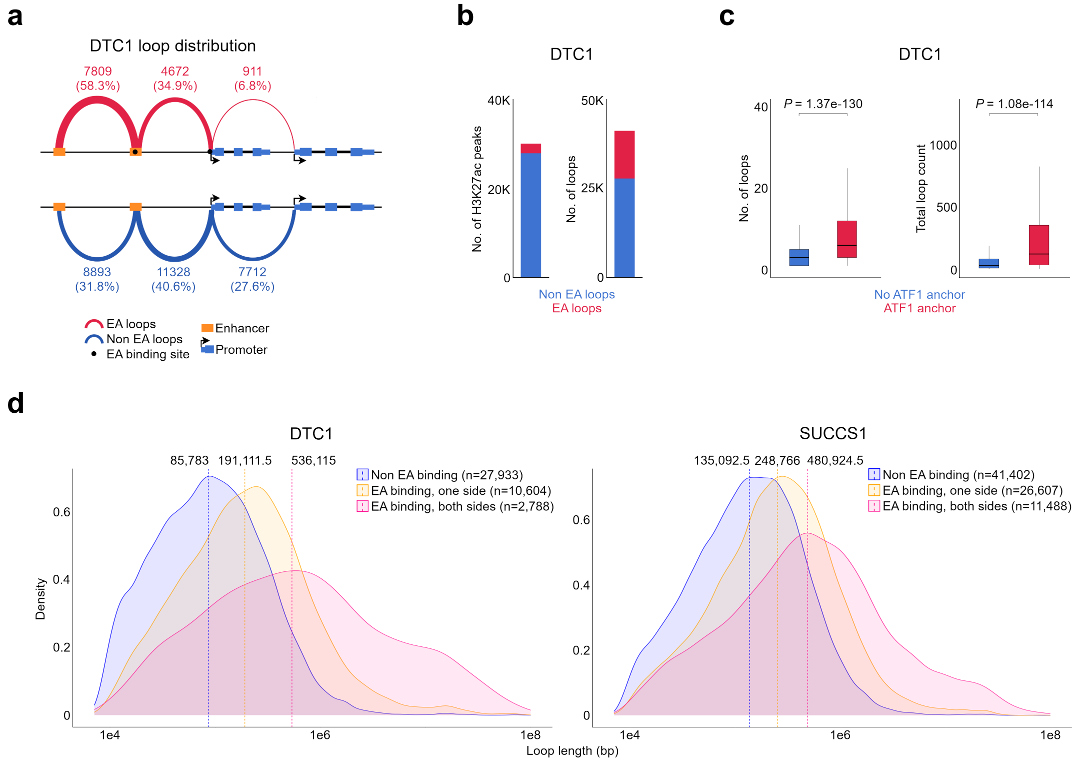

Supplementary Fig. 5: EWSR1-ATF1-associated chromatin interactions dominates the 3D connectivity landscape of CCS tumor cells.

a, Three-dimensional connectivity of EWSR1-ATF1-associated (EA loops, red) or –independent (Non-EA loops, blue) chromatin loops in DTC1 tumor cells, showing a more prevalent distal-to-distal pattern of interaction for fusion protein-connected loops. **b,** Bar plots showing the association of H3K27ac peaks (*left*) and chromatin loops (*right*) with EA loops (2323 and 13392, respectively) or Non-EA loops (28031 and 27933, respectively). **c,** Box plots depicting the median number (*left*) and intensity (*right*) of chromatin loops associated (n= 13392) or not (n= 27933) with EWSR1-ATF1 binding sites. The lower and upper hinges correspond to the first and third quartiles (the 25th and 75th percentiles). The upper whisker extends from the hinge to the largest value no further than 1.5 * IQR from the hinge (where IQR is the inter-quartile range). The lower whisker extends from the hinge to the smallest value at most 1.5 * IQR of the hinge. Statistical significance was calculated by two-sided t-test (n= 1863 for ATF1 anchors, n= 17041 for No ATF1 anchors). **d,** Density plots showing the differences in length between EWSR1-ATF1-associated (EA binding, orange and pink) or -independent (Non-EA binding, blue) loops genome-wide. In both DTC1 and SU-CCS-1 cell lines the presence of the fusion protein is associated with increases in loop length.

Supplementary Fig. 6

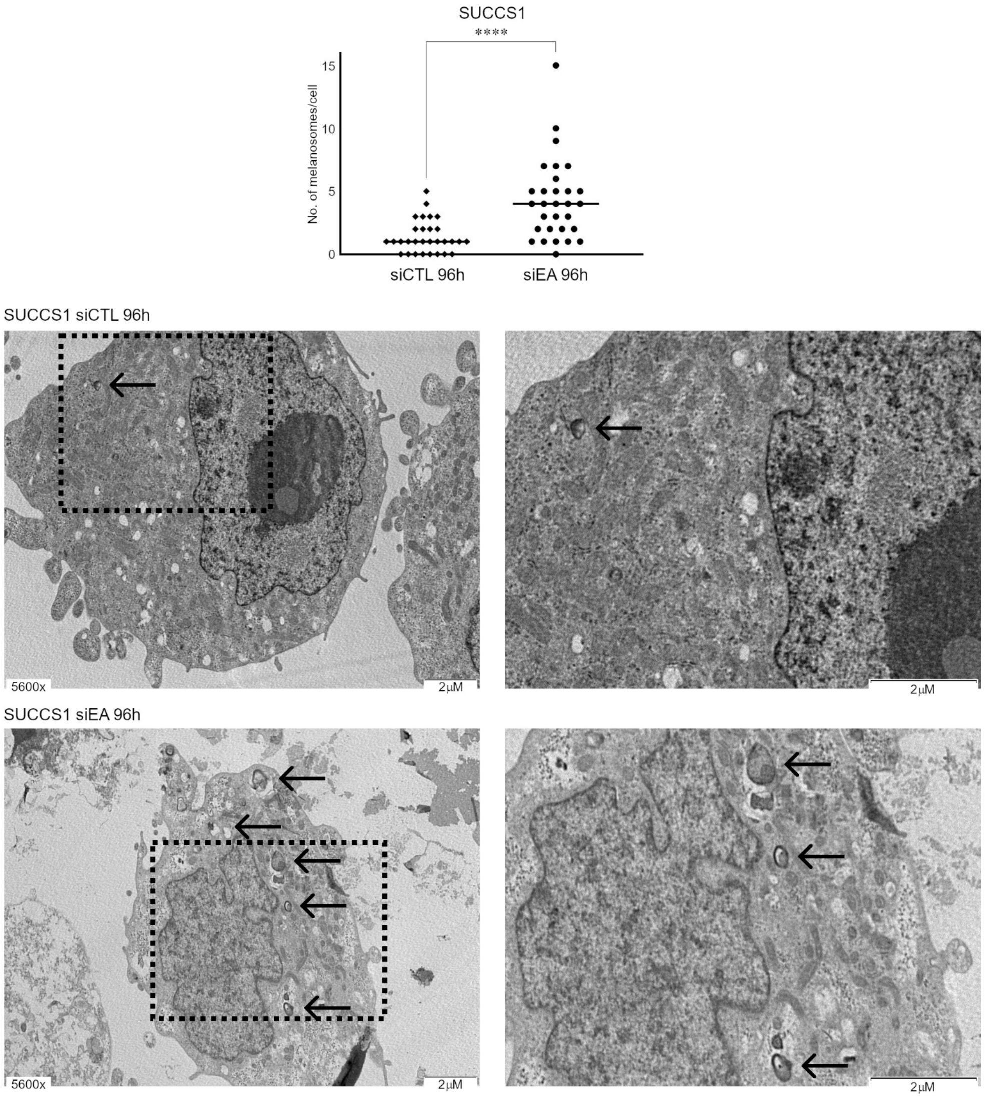

Supplementary Fig. 6: EWSR1-ATF1 depletion increases the number of melanosomes in SU-CCS-1 cells. *Top panel*: Melanosome counts/cell in SU-CCS-1 cells treated with control (siCTL) or *EWSR1-ATF1* targeting (siEA) siRNAs for 96h. 30 cells/condition were surveyed, horizontal bars show mean values. Statistical significance was calculated by two-sided t-test (**** = p-value < 0.0001, exact p-value 4,83e-05). *Bottom panel*: Electron microscopy images of representative cells for each condition at 5600x magnification (left). Dashed line rectangles indicate the area shown at higher magnification in the right panels. Arrows indicate melanosomes. Source data are provided in the Source Data file.

Supplementary Table 1. Primers used for RQ-PCR and cloning.

Primer name	Sequence (5' – 3')	Used for
IRF4_F	GGGCAAGCAGGACTACAAC	RQ-PCR
IRF4_R	GGGTCCGGCTTGTCGATG	RQ-PCR
CDK2_F	ATGGATGCCTCTGCTCTCACTG	RQ-PCR
CDK2_R	CCCGATGAGAATGGCAGAAAGC	RQ-PCR
cMET_F	AGCAATGGGGAGTGTAAAGAGG	RQ-PCR
cMET_R	CCCAGTCTTGTACTCAGCAAC	RQ-PCR
ERBB3_F	ACCAGTGCTGCCATGATGAG	RQ-PCR
ERBB3_R	GCTCCACTGTCATTGAAGTGC	RQ-PCR
MERTK_F	GATCGTGTCTGATCCCATCTAC	RQ-PCR
MERTK_R	GAAGGCTGTGTTTCTGGTGAC	RQ-PCR
OCA2_F	GGCCAGGAGTTTGCTTCATTC	RQ-PCR
OCA2_R	ATGCACCGTGACCCTTTCTC	RQ-PCR
PMEL_F	CTCATTCCAGCTCAGCCTTC	RQ-PCR
PMEL_R	AAGTGCTTGTTCCCTCCATC	RQ-PCR
EWSR1[1]_1265F	TTTGATCGTGGAGGCATGAG	RQ-PCR (EWSR1-ATF1)
ATF1[2]_585R	TGACAGCAGCAGAAACTCCAG	RQ-PCR (EWSR1-ATF1)
ATF1_334F	CGTCAGAGACAGCACCTCAAC	RQ-PCR (wt ATF1)
ATF1_455R	GCCTATGCTGTCGGATGAGTC	RQ-PCR (wt ATF1)
GAPDH_F	GGTCTCCTCTGACTTCAACA	RQ-PCR (control)
GAPDH_R	GTGAGGGTCTCTCTCTTCCT	RQ-PCR (control)
EWSR1_269F	CGCCTAGAGGGAAAGCGAGA	Cloning (EA CDS)
ATF1_1313R	CCCGTAGCTTCCTTGAGGTC	Cloning (EA CDS)
EWSR1_296F	CGGACGTTGAGAGAACGAGGAG	Cloning (EA CDS)
ATF1_1245R	TCCTTGATTTGGAAAAGCCTAA	Cloning (EA CDS)
EWSR1_LR_F	CACCATGGCGTCCACGGATTACAGTACCT	Cloning (NV5 EA)
ATF1_V5_LR_R	GCGTCACGTAGAATCGAGACCGAGGAGAGGGTTAGGGATAGGCTTACCAACACTTTTATTGG	Cloning (NV5 EA)
EWSR1_V5_LR_F	CACCATGGGTAAGCCTATCCCTAACCCTCTCCTCGGTCTCGATTCTACGGCGTCCACGGATT	Cloning (CV5 EA)
ATF1_LR_R	GCGTCAAACACTTTTATTGGAATAAAGATCCTTCAAAGTTTTTAACTC	Cloning (CV5 EA)
ATF1_216F	GTGGGGAAGTGGGTAGTGA	Cloning (ATF1 CDS)
ATF1_1493R	TCAGCAAACAAATGCAAACC	Cloning (ATF1 CDS)
ATF1_LR_F	CACCATGGAAGATTCCCACAAGAGTACCAC	Cloning (ATF1 CV5)
EWSR1_mut_ATF1_F	CACCGCCACCATGGCCAGTACAGAC	Cloning (YS37mutant)
EWSR1_mut_ATF1_R	TCAGCTCGCATAATCCGGTACGTCATACGGGTAG	Cloning (YS37mutant)
EWSR1[1]_1265F	TTTGATCGTGGAGGCATGAG	RQ-PCR (EWSR1-ATF1)
ATF1[2]_585R	TGACAGCAGCAGAAACTCCAG	RQ-PCR (EWSR1-ATF1)
ATF1_334F	CGTCAGAGACAGCACCTCAAC	RQ-PCR (wt ATF1)
ATF1_455R	GCCTATGCTGTCGGATGAGTC	RQ-PCR (wt ATF1)
GAPDH_F	GGTCTCCTCTGACTTCAACA	RQ-PCR (control)
GAPDH_R	GTGAGGGTCTCTCTCTTCCT	RQ-PCR (control)
EWSR1_269F	CGCCTAGAGGGAAAGCGAGA	Cloning (EA CDS)
ATF1_1313R	CCCGTAGCTTCCTTGAGGTC	Cloning (EA CDS)
EWSR1_296F	CGGACGTTGAGAGAACGAGGAG	Cloning (EA CDS)
ATF1_1245R	TCCTTGATTTGGAAAAGCCTAA	Cloning (EA CDS)
EWSR1_LR_F	CACCATGGCGTCCACGGATTACAGTACCT	Cloning (NV5 EA)
ATF1_V5_LR_R	GCGTCACGTAGAATCGAGACCGAGGAGAGGGTTAGGGATAGGCTTACCAACACTTTTATTGG	Cloning (NV5 EA)
EWSR1_V5_LR_F	CACCATGGGTAAGCCTATCCCTAACCCTCTCCTCGGTCTCGATTCTACGGCGTCCACGGATT	Cloning (CV5 EA)
ATF1_LR_R	GCGTCAAACACTTTTATTGGAATAAAGATCCTTCAAAGTTTTTAACTC	Cloning (CV5 EA)
ATF1_216F	GTGGGGAAGTGGGTAGTGA	Cloning (ATF1 CDS)
ATF1_1493R	TCAGCAAACAAATGCAAACC	Cloning (ATF1 CDS)

[1]EWSR1 reference sequence NM_013986, [2]ATF1 reference sequence NM_005171

Supplementary Information Source Data

For original western blot images: Membranes were cut for the primary antibody incubations. Framed sections came from the same membrane. Red frames indicate the cropped image used for the figure.

Supplementary Fig. 1c: DTC1 EWSR1-ATF1 : BRG1 co-IP

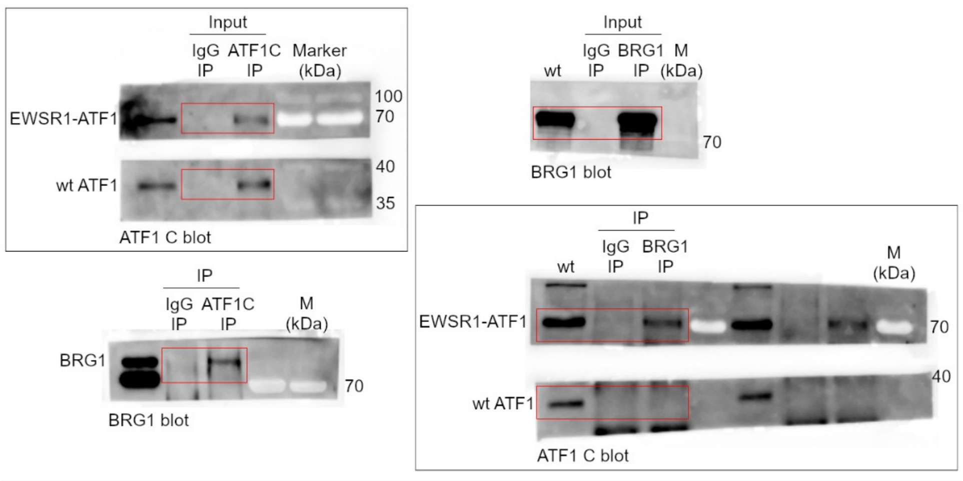

Supplementary Fig. 2a: siRNA depletion of EWSR1-ATF1

Sample name	Target Name	Ct Mean	Ct SD
DTC1 siEA 72h	GAPDH	17,38	0,13
DTC1 siEA 96h	GAPDH	19,52	0,22
DTC1 siCTL 96h	GAPDH	17,15	0,07
DTC1 siEA 72h	5'ATF1	24,51	0,13
DTC1 siEA 96h	5'ATF1	26,68	0,06
DTC1 siCTL 96h	5'ATF1	24,38	0,09
DTC1 siEA 72h	EWSR1-ATF1	33,60	0,31
DTC1 siEA 96h	EWSR1-ATF1	37,09	0,08
DTC1 siCTL 96h	EWSR1-ATF1	32,17	0,06

Sample name	Target Name	Ct Mean	Ct SD
SU-CCS-1 siEA 72h	GAPDH	16,88	0,09
SU-CCS-1 siEA 96h	GAPDH	16,89	0,05
SU-CCS-1 siCTL 96h	GAPDH	16,88	0,15
SU-CCS-1 siEA 72h	5'ATF1	23,20	0,08
SU-CCS-1 siEA 96h	5'ATF1	23,22	0,05
SU-CCS-1 siCTL 96h	5'ATF1	23,03	0,06
SU-CCS-1 siEA 72h	EWSR1-ATF1	33,59	0,07
SU-CCS-1 siEA 96h	EWSR1-ATF1	34,44	0,06
SU-CCS-1 siCTL 96h	EWSR1-ATF1	30,18	0,19

Supplementary Fig. 4a, d, e, f and g: EWSR1-ATF1 constructs over expression in hpMSCs.

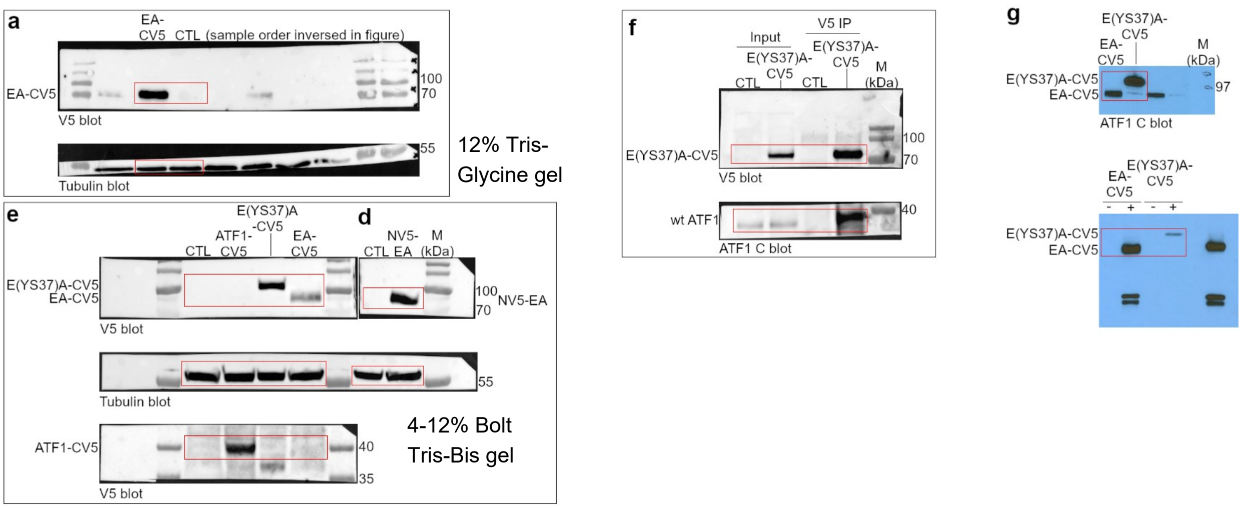

Supplementary Fig. 6. Melanosome count in siEA- and siCTL treated SU-CCS-1.

SU-CCS-1 siCTL		SU-CCS-1 siEA	
Cell number	Melanosome count	Cell number	Melanosome count
1	5	1	5
2	0	2	3
3	2	3	1
4	2	4	4
5	0	5	6
6	1	6	1
7	1	7	2
8	3	8	1
9	1	9	0
10	0	10	5
11	1	11	3
12	3	12	3
13	0	13	10
14	1	14	7
15	2	15	7
16	1	16	15
17	1	17	4
18	0	18	4
19	1	19	2
20	0	20	5
21	1	21	2
22	1	22	5
23	2	23	1
24	1	24	4
25	3	25	7
26	0	26	9
27	1	27	2
28	3	28	1
29	4	29	5
30	0	30	4
