# Supplementary material for: EWSR1-ATF1 dependent 3D connectivity regulates oncogenic and differentiation programs in Clear Cell Sarcoma
Source: Nat Commun. 2022 Apr 27;13:2267. doi: 10.1038/s41467-022-29910-4 (PMC9046276; doi:10.1038/s41467-022-29910-4)
Supplement: Supplementary file 3 — Description of Additional Supplementary Files [file 41467_2022_29910_MOESM3_ESM.pdf]

Description of Additional Supplementary Files

File Name: Supplementary Data 1

Description:

Tab1: Common Hi-ChIP targets. List of 2014 EWSR1-ATF1 direct target genes (column A: gene symbol, column B: Ensembl ID) shared between SU-CCS-1 and DTC1 cells, and identified by H3K27ac Hi-ChIP analysis. The results from the RNA-seq differential gene expression analysis of SU-CCS-1 and DTC1 cells treated with siRNAs targeting EWSR1-ATF1 (siEA) vs control (siCTL) are shown in column C (log FC = log fold change values) and D (Bejamini Hochberg corrected p-values).

Tab2: Hi-ChIP targets GO analysis. Functional analysis of 2014 common HiChIP target genes for Canonical Pathways (CP:Biocarta, CP:KEGG, CP:PID, CP:Reactome and CP:Wikipathways) and Gene Ontologies Biological processes (GO:BP), Cellular component (GO:CC) and Molecular Function (GO:MF).

Tab3: HiChIP targets DEG. List of the HiChIP direct targets genes that were differentially expressed in siEA-treated SU-CCS-1 and DTC1 cells at FC=1.5 (n=535) (column A: gene symbol, column B: Ensembl ID, column C: log FC values and column D: adjusted p-values), the majority of which (n=417) were downregulated.

Tab4: Downregulated HiChIP targets GO analysis. Functional analysis of the 417 downregulated HiChIP target genes in siEA-treated cells for Canonical Pathways (CP:Biocarta, CP:KEGG, CP:PID, CP:Reactome and CP:Wikipathways) and the Gene Ontologies Biological process (GO:BP), Cellular component (GO:CC) and Molecular Function (GO:MF), showing an enrichment for several cell cycle related functions and processes.

Tab5: siEA de novo H3K27ac targets. List of the direct target genes of the "*de novo*" H3K27ac sites in siEA-treated SU-CCS-1 and DTC1 cells that were also differentially upregulated in siEA-treated cells at cutoffs FC

1.5 and p-value 0.05 (n=157) (column A: gene symbol, column B: Ensembl ID, column C: log FC values and column D: adjusted p-values).

Tab6: de novo upregulated targets GO analysis. Functional analysis of the 157 upregulated ”*de novo”* target genes for Canonical Pathways (CP:Biocarta, CP:KEGG, CP:PID, CP:Reactome and CP:Wikipathways) and the Gene Ontologies Biological processes (GO:BP), Cellular component (GO:CC) and Molecular Function (GO:MF), showing enrichment for several functions associated with cell differentiation and chromatin regulation.
