# Supplementary material for: EWSR1-ATF1 dependent 3D connectivity regulates oncogenic and differentiation programs in Clear Cell Sarcoma
Source: Nat Commun. 2022 Apr 27;13:2267. doi: 10.1038/s41467-022-29910-4 (PMC9046276; doi:10.1038/s41467-022-29910-4)
Supplement: Supplementary file 5 — Reporting Summary [file 41467_2022_29910_MOESM5_ESM.pdf]

nature portfolio

Corresponding author(s): Nicolò Riggi
Nicolo.Riggi@chuv.ch

Last updated by author(s): 2022.03.03

Reporting Summary

Nature Portfolio wishes to improve the reproducibility of the work that we publish. This form provides structure for consistency and transparency in reporting. For further information on Nature Portfolio policies, see our Editorial Policies and the Editorial Policy Checklist.

Statistics

For all statistical analyses, confirm that the following items are present in the figure legend, table legend, main text, or Methods section.

n/a	Confirmed	
☐	☒	The exact sample size (*n*) for each experimental group/condition, given as a discrete number and unit of measurement
☐	☒	A statement on whether measurements were taken from distinct samples or whether the same sample was measured repeatedly
☐	☒	The statistical test(s) used AND whether they are one- or two-sided<br>*Only common tests should be described solely by name; describe more complex techniques in the Methods section.*
☐	☒	A description of all covariates tested
☐	☒	A description of any assumptions or corrections, such as tests of normality and adjustment for multiple comparisons
☐	☒	A full description of the statistical parameters including central tendency (e.g. means) or other basic estimates (e.g. regression coefficient) AND variation (e.g. standard deviation) or associated estimates of uncertainty (e.g. confidence intervals)
☐	☒	For null hypothesis testing, the test statistic (e.g. *F*, *t*, *r*) with confidence intervals, effect sizes, degrees of freedom and *P* value noted<br>*Give P values as exact values whenever suitable.*
☒	☐	For Bayesian analysis, information on the choice of priors and Markov chain Monte Carlo settings
☒	☐	For hierarchical and complex designs, identification of the appropriate level for tests and full reporting of outcomes
☒	☐	Estimates of effect sizes (e.g. Cohen's *d*, Pearson's *r*), indicating how they were calculated

Our web collection on statistics for biologists contains articles on many of the points above.

Software and code

Policy information about availability of computer code

Data collection	External data was acquired using fastq-dump from the SRA toolkit version 2.10.3 when raw files were used. Otherwise, no software was used.
Data analysis	Raw HTS reads were trimmed using Trimgalore v 0.6.4 and aligned to the hg19 genome/transctiptome using STAR v 2.7.3a.<br>For ChIPseq, peaks were first called with MACS2 v 2.2.6 with an FDR of 1e-7 for ATF1 C, ATF1 N, H3K4me3 and V5, and 1e-4 for EWSR1, using the corresponding input samples. MACS peaks were then subjected to a second round of selection as described in Methods. CPM-normalized BigWig files were generated using bamCoverage and used for the ChIP signal heat maps and composite plots (generated with the DeepTools v 3.5.0 scripts computeMatrix, plotHeatmap and plotProfile). ChIP peaks scores were calculated using bedtools with option -c normalized first per library to 20M reads and then per peak length to 500 bp. The final peak score (S) is given by S=log2(IP+1)-log2(Input+1)), where +1 is one additional pseudocount. HOMER v 4.11.1 was used for ChIP-seq peak motif enrichment analysis.<br>For Hi-ChIP seq, paired-end reads were aligned to the hg19 genome using the HiC-Pro pipeline v 2.7.6 and default settings. The hichipper tool v 0.7.7 was used for loop calling by H3K27ac ChIP-seq peaks (q-value <= 0.00001), applying the parameters; max distance 100000000, read length 100. Differential loops between siEA and siCTL treated cells, loops were normalized using DESeq2 and analyzed using the DiffLoop tool.<br>For RNA-seq, gene counts were computed using using rsem v 1.3.0. The counts matrix was then processed in R for differential analysis (using EdgeR package v 3.32.1, Limma voom and removeBatchEffect functions and RUVseq package v 1.24.0), as explained in Methods.<br>For the external scRNA-seq data, the provided counts and cells annotations were analyzed using the following R packages: Seurat_4.0.2, SeuratDisk_0.0.0.9019, zellkonverter_1.2.0, SeuratObject_4.0.1 and dittoSeq_1.4.1.<br>All other figures were generated using R v 4.1.0. No previously unreported custom computer codes or algorithms were used to generate the results reported in this paper.

For manuscripts utilizing custom algorithms or software that are central to the research but not yet described in published literature, software must be made available to editors and reviewers. We strongly encourage code deposition in a community repository (e.g. GitHub). See the Nature Portfolio guidelines for submitting code & software for further information.

Data

Policy information about availability of data

All manuscripts must include a data availability statement. This statement should provide the following information, where applicable:

- Accession codes, unique identifiers, or web links for publicly available datasets
- A description of any restrictions on data availability
- For clinical datasets or third party data, please ensure that the statement adheres to our policy

The raw data generated in this study are publicly available at the Gene Expression Omnibus (GEO) database under the series accession number GSE180198 [https://www.ncbi.nlm.nih.gov/geo/query/acc.cgi?acc=GSE180198] which contains the datasets GSE180183 (ATAC-seq), GSE180187 (ChIP-seq), GSE180194 (HiChIP-seq) and GSE180196 (RNA-seq). The human genome sequence data (GRCh37, hg19) used in this study is available through the Ensembl genome browser [https://grch37.ensembl.org/Homo_sapiens/Info/Index]. The wt ATF1 ChIP-seq data used in this study are available through the ENCODE database [https://www.encodeproject.org/experiments/ENCSR253OON/, https://www.encodeproject.org/experiments/ENCSR091GVJ/]. The DNAse-seq profiles used in this study are available through the GEO database [https://www.ncbi.nlm.nih.gov/geo/query/acc.cgi?acc=GSE29692]. The single cell RNAseq data used in this study are available from ZENODO [https://zenodo.org/record/4569496#.YMdnr5ozZYg]. The remaining data available within the Article, Supplementary Information or Source Data file.

Field-specific reporting

Please select the one below that is the best fit for your research. If you are not sure, read the appropriate sections before making your selection.

- [x] Life sciences
- [ ] Behavioural & social sciences
- [ ] Ecological, evolutionary & environmental sciences

For a reference copy of the document with all sections, see nature.com/documents/nr-reporting-summary-flat.pdf

Life sciences study design

All studies must disclose on these points even when the disclosure is negative.

Sample size	Sample size selection was based on availability (no. of primary samples) and to provide sufficient statistical power to identify significant differences (providing replicates when feasible), given our previous knowledge about sample variation for the respective technique. Sample sizes were not pre-defined based on statistical methods.
Data exclusions	None of the data were excluded from the study. The data selection criteria for each downstream analysis are stated in the manuscript.
Replication	To ensure a solid identification of the EWSR1-ATF1 reference peak set by ChIP-seq, two replicates of each antibody was performed for both wt cell lines DTC1 and SU-CCS-1. For the other experiments, one replicate was used for the analysis. To ensure reproducibility, all experiments except Hi-ChIPseq experiments were independently repeated twice generating similar results. Due to limited availability of primary hpMSCs, the EWSR1(YS37)-ATF1 mutant over expression ChIPseq experiment could only be performed once.
Randomization	Randomization was not relevant to the experiments of this study as effects on experimental groups were not compared. For the transcriptomic analyses, the global differences between sample groups (where randomization could have been applied) was not analyzed. The transcriptome data were only used to look at expression levels of pre-defined gene lists.
Blinding	All the data of the study were analyzed without pre-established conclusions. Bioinformaticians were blinded to any sample type or group allocations during data collection.

Reporting for specific materials, systems and methods

We require information from authors about some types of materials, experimental systems and methods used in many studies. Here, indicate whether each material, system or method listed is relevant to your study. If you are not sure if a list item applies to your research, read the appropriate section before selecting a response.

Materials & experimental systems

n/a	Involved in the study	
[ ]	[x]	Antibodies
[ ]	[x]	Eukaryotic cell lines
[x]	[ ]	Palaeontology and archaeology
[x]	[ ]	Animals and other organisms
[ ]	[x]	Human research participants
[x]	[ ]	Clinical data
[x]	[ ]	Dual use research of concern

Methods

n/a	Involved in the study	
[ ]	[x]	ChIP-seq
[x]	[ ]	Flow cytometry
[x]	[ ]	MRI-based neuroimaging

Antibodies

Antibodies used	Anti-ATF1 C (1:500, cat#LS-C351857, LS Bio, lot#166109, or ab134104, Abcam, lot#GR97084-8), -ATF1 N (1:2000, cat#A303-034A, Bethyl laboratories, lot#2), -EWSR1 N (C-9, cat#sc-48404, Santa Cruz, lot#J2517), -TFAP2A (3B5, cat#sc-12726, Santa Cruz, lot#H1220), -SOX10 (cat#PA5-40697, Invitrogen, lot#WH3347801), -MITF (cat#91201, Active motif, lot#30918002), -H3K4me3 (cat#07-473, Millipore, lot#2648189), -H3K27ac (cat#39133, Active motif, lot#31814008), -H3K4me1 (cat#ab8895, Abcam, lot#GR159018-1), -p300 (D2X6N, cat#54062, Cell signaling), -V5 (for ChIPseq; D3H8Q, cat#13202, Cell signaling, lot#5, for western blot; 1:5000, cat#46-1157, Invitrogen, lot#1965106), -Tubulin (1:1000, DM1A, cat#CP06, Millipore, lot#3313101), -BRG1 (1:1000, cat#E906E, Cell signaling, lot#1) and HRP-conjugated sheep anti-mouse (1:5000, cat#NA931V, Cytiva, lot#17016967) or goat anti-rabbit (1:20 000, cat#P0448, Dako, lot#20083037) secondary antibodies. Where antiody dilutions are not indicated, 1ug antibody/million cells was used.
Validation	The ATF1 C and ATF1 N antibodies were verified by western blot and/or ChIPseq of ATF1 KD vs control cells, and V5 antibodies by western blot and ChIPseq of V5 construct overexpression vs control cells. The other antibodies have been validated by our previous work (anti-H3K4me3 and -H3K27ac, PMID: 25453903) or by the manufacturers´ for the application used;<br>https://www.lsbio.com/antibodies/atf1-antibody-c-terminus-icc-if-immunofluorescence-ihc-ip-wb-western-ls-c351857/362978<br>https://www.fortislife.com/products/primary-antibodies/rabbit-anti-atf1-antibody/BETHYL-A303-034<br>https://www.scbt.com/p/ews-antibody-c-9<br>https://www.cellsignal.com/products/primary-antibodies/brg1-e9o6e-mouse-mab/52251 (cross-species reactivity)<br>https://www.cellsignal.com/products/primary-antibodies/v5-tag-d3h8q-rabbit-mab/13202 (cross-species reactivity)<br>https://www.scbt.com/p/ap-2alpha-antibody-3b5,<br>https://www.thermofisher.com/antibody/product/SOX10-Antibody-Polyclonal/PA5-40697,<br>https://www.activemotif.com/catalog/details/91201/abflex-mitf-antibody-rab,<br>https://www.abcam.com/Histone-H3-mono-methyl-K4-antibody-ChIP-Grade-ab8895,<br>https://www.cellsignal.com/products/primary-antibodies/p300-d2x6n-rabbit-mab/54062,<br>https://www.merckmillipore.com/CH/de/product/Anti-Tubulin-Mouse-mAb-DM1A,EMD_BIO-CP06,<br>https://www.citeab.com/antibodies/3288287-na931-1ml-amersham-ecl-mouse-igg-hrp-linked-whole-a,<br>https://www.citeab.com/antibodies/3288347-p0448-goat-anti-rabbit-immunoglobulins-hrp-affinity<br>Anti-V5 tag antibody 46-1157 is available under an updated catalog number. Antibody citation: DOI:10.1371/journal.pone.0188311

Eukaryotic cell lines

Policy information about cell lines

Cell line source(s)	HEK293T and SU-CCS-1 were obtained from ATCC. DTC1 was provided by Professor T. Nielsen (Department of Pathology and Laboratory Medicine, University of British Columbia, Vancouver, Canada). Citation for DTC1: Brown, A D. et al. Promoters containing ATF-binding sites are de-regulated in cells that express the EWS/ATF1 oncogene. Oncogene 10, 1749-56, (1995), PMID:7753552.
Authentication	We periodically evaluated the DTC1 and SU-CCS-1 cell lines for EWSR1-ATF1 expression by PCR (RT-PCR, RQ-PCR) using specific primers followed by Sanger sequencing of purified fusion transcript cDNA. Presence of t(12;22)(q13;q12) was verified by karyotyping. The cell lines were not authenticated by other methods.
Mycoplasma contamination	All cell cultures were periodically tested negative for mycoplasma contamination by PCR-based methods.
Commonly misidentified lines (See ICLAC register)	No commonly misidentified cell lines were used.

Human research participants

Policy information about studies involving human research participants

Population characteristics	Donors were of no specific population, samples were collected from anonymous individuals.
Recruitment	Primary tumor samples and healthy bone marrow samples (for hpMSC isolation) were obtained under informed consent. Participants were chosen completely randomly and de-identified prior to being obtained and analyzed.
Ethics oversight	Samples were collected with approval from the Institutional review boards of Centre Hospitalier Universitaire Vaudois (CHUV, University of Lausanne, Switzerland), Massachusetts General Hospital (MGH, Boston, USA) and Skåne University Hospital (Lund University, Sweden). Samples were anonymized prior to analysis. Informed consent was obtained for prospective samples.

Note that full information on the approval of the study protocol must also be provided in the manuscript.

ChIP-seq

Data deposition

- [x] Confirm that both raw and final processed data have been deposited in a public database such as GEO.
- [x] Confirm that you have deposited or provided access to graph files (e.g. BED files) for the called peaks.

Data access links
May remain private before publication.

https://www.ncbi.nlm.nih.gov/geo/query/acc.cgi?acc=gse180198

Files in database submission

C_1_1_SUCCS1_wt_H3K27ac_1.fastq.gz
C_1_3_SUCCS1_wt_H3K4me3_1.fastq.gz
C_1_4_SUCCS1_wt_H3K4me1_1.fastq.gz
C_1_8_SUCCS1_wt_ATF1_C_1.fastq.gz
C_1_9_SUCCS1_wt_Input_1.fastq.gz
C_1_10_SUCCS1_wt_H3K27ac_2.fastq.gz
C_1_12_SUCCS1_wt_H3K4me3_2.fastq.gz
C_1_13_SUCCS1_wt_H3K4me1_2.fastq.gz
C_1_17_SUCCS1_wt_ATF1_C_2.fastq.gz
C_1_21_SUCCS1_wt_ATF1_N_2.fastq.gz
C_1_20_SUCCS1_wt_Input_2.fastq.gz
C_1_19_SUCCS1_wt_ATF1_N_1.fastq.gz
C_1_23_SUCCS1_wt_Input_3.fastq.gz
C_14_5_SUCCS1_wt_EWSR1_2.fastq.gz
C_14_6_SUCCS1_wt_EWSR1_3.fastq.gz
C_3_1_DTC1_wt_H3K27ac_1.fastq.gz
C_3_3_DTC1_wt_H3K4me3_1.fastq.gz
C_3_4_DTC1_wt_H3K4me1_1.fastq.gz
C_3_7_DTC1_wt_ATF1_C_1.fastq.gz
C_3_8_DTC1_wt_ATF1_N_1.fastq.gz
C_3_9_DTC1_wt_Input_1.fastq.gz
C_3_10_DTC1_wt_H3K27ac_2.fastq.gz
C_3_12_DTC1_wt_H3K4me3_2.fastq.gz
C_3_13_DTC1_wt_H3K4me1_2.fastq.gz
C_3_16_DTC1_wt_ATF1_C_2.fastq.gz
C_3_17_DTC1_wt_ATF1_N_2.fastq.gz
C_3_18_DTC1_wt_Input_2.fastq.gz
C_14_3_DTC1_wt_EWSR1_2.fastq.gz
C_14_4_DTC1_wt_EWSR1_3.fastq.gz
C_5_1_MSC_2_empty_H3K27ac_1.fastq.gz
C_5_2_MSC_2_empty_V5_1.fastq.gz
C_5_3_MSC_2_empty_ATF1_N_1.fastq.gz
C_5_4_MSC_2_empty_Input_1.fastq.gz
C_5_5_MSC_2_EACTD_H3K27ac_1.fastq.gz
C_5_6_MSC_2_EACTD_V5_1.fastq.gz
C_5_7_MSC_2_EACTD_ATF1_N_1.fastq.gz
C_5_8_MSC_2_EACTD_Input_1.fastq.gz
C_5_9_MSC_2_EANTD_H3K27ac_1.fastq.gz
C_5_10_MSC_2_EANTD_V5_1.fastq.gz
C_5_11_MSC_2_EANTD_ATF1_N_1.fastq.gz
C_5_12_MSC_2_EANTD_Input_1.fastq.gz
C_7_1_DTC1_siCTL_96h_H3K27ac_1.fastq.gz
C_7_2_DTC1_siCTL_96h_ATF1_C_1.fastq.gz
C_7_3_DTC1_siCTL_96h_ATF1_N_1.fastq.gz
C_7_4_DTC1_siCTL_96h_Input_1.fastq.gz
C_7_9_DTC1_siEA_96h_H3K27ac_1.fastq.gz
C_7_10_DTC1_siEA_96h_ATF1_C_1.fastq.gz
C_7_11_DTC1_siEA_96h_ATF1_N_1.fastq.gz
C_7_12_DTC1_siEA_96h_Input_1.fastq.gz
C_7_13_DTC1_siCTL_96h_H3K4me1_1.fastq.gz
C_7_14_DTC1_siCTL_96h_H3K4me3_1.fastq.gz
C_7_19_DTC1_siEA_96h_H3K4me1_1.fastq.gz
C_7_20_DTC1_siEA_96h_H3K4me3_1.fastq.gz
C_10_1_SUCCS1_siCTL_96h_H3K4me3_1.fastq.gz
C_10_2_SUCCS1_siCTL_96h_H3K27ac_1.fastq.gz
C_10_4_SUCCS1_siCTL_96h_Input_1.fastq.gz
C_10_5_SUCCS1_siCTL_96h_ATF1_C_1.fastq.gz

C_10_6_SUCCS1_siCTL_96h_ATF1_N_1.fastq.gz
C_10_11_SUCCS1_siEA_96h_H3K4me3_1.fastq.gz
C_10_12_SUCCS1_siEA_96h_H3K27ac_1.fastq.gz
C_10_13_SUCCS1_siEA_96h_ATF1_C_1.fastq.gz
C_10_14_SUCCS1_siEA_96h_ATF1_N_1.fastq.gz
C_10_15_SUCCS1_siEA_96h_Input_1.fastq.gz
C_10_16_SUCCS1_siEA_96h_H3K4me1_1.fastq.gz
C_11_1_SUCCS1_wt_p300_1.fastq.gz
C_11_5_DTC1_wt_p300_1.fastq.gz
C_11_6_MSC_2_EACTD_p300_1.fastq.gz
C_11_7_MSC_2_EANTD_p300_1.fastq.gz
C_11_8_MSC_2_empty_p300_1.fastq.gz
C_13_16_SUCCS1_siCTL_96h_H3K4me1_1.fastq.gz
Primary_AFH1_H3K27ac_1.fastq.gz
Primary_AFH1_H3K4me1_1.fastq.gz
Primary_AFH1_H3K4me3_1.fastq.gz
Primary_AFH1_Input_1.fastq.gz
Primary_AFH2_H3K27ac_1.fastq.gz
Primary_AFH2_H3K4me1_1.fastq.gz
Primary_AFH2_H3K4me3_1.fastq.gz
Primary_AFH2_Input_1.fastq.gz
Primary_CCS2_H3K27ac_1.fastq.gz
Primary_CCS2_H3K4me1_1.fastq.gz
Primary_CCS2_H3K4me3_1.fastq.gz
Primary_CCS2_Input_1.fastq.gz
Primary_CCS1_ATF1_C_1.fastq.gz
Primary_CCS1_ATF1_N_1.fastq.gz
Primary_CCS1_H3K27ac_2.fastq.gz
Primary_CCS1_H3K4me1_2.fastq.gz
Primary_CCS1_H3K4me3_2.fastq.gz
Primary_CCS1_Input_1.fastq.gz
Primary_CCS3_H3K27ac_1.fastq.gz
Primary_CCS3_H3K4me1_1.fastq.gz
Primary_CCS3_H3K4me3_1.fastq.gz
Primary_CCS3_Input_1.fastq.gz
SUCCS1_wt_TFAP2A_1.fastq.gz
DTC1_wt_TFAP2A_1.fastq.gz
MSC_2_ATF1_CTD_V5_1.fastq.gz
MSC_2_EACTD_H3K4me3_2.fastq.gz
MSC_2_EACTD_V5_2.fastq.gz
MSC_2_EA_YS37mut_CTD_H3K4me3_1.fastq.gz
MSC_2_EA_YS37mut_CTD_V5_1.fastq.gz
C_5_14_MSC_2_empty_H3K4me3_1.fastq.gz
DTC1_WT_MITF.fastq.gz
DTC1_WT_SOX10.fastq.gz
SUCCS1_shATF1_ATF1_C.fastq.gz
SUCCS1_shATF1_ATF1_N.fastq.gz
SUCCS1_shATF1_H3K27ac.fastq.gz
SUCCS1_shCTRL_ATF1_C.fastq.gz
SUCCS1_shCTRL_ATF1_N.fastq.gz
SUCCS1_shCTRL_H3K27ac.fastq.gz
SUCCS1_siCTRL_MITF.fastq.gz
SUCCS1_siCTRL_SOX10.fastq.gz
SUCCS1_siCTRL_TFAP2A.fastq.gz
SUCCS1_siEA_MITF.fastq.gz
SUCCS1_siEA_SOX10.fastq.gz
SUCCS1_siEA_TFAP2A.fastq.gz
SUCCS1_WT_MITF.fastq.gz
SUCCS1_WT_SOX10.fastq.gz

Genome browser session
(e.g. UCSC)

No longer applicable.

Methodology

Replicates

For DTC1 and SU-CCS-1 wt cell lines ChIP-seq, two replicates of each cell line and each antibody was performed. For the other ChIPseq experiments, one replicate per sample was sequenced.

Sequencing depth	All ChIPseq samples were sequenced on an Illumina HiSeq 4000 as 50bp single-end reads, and all HiChIPseq samples as 100bp paired-end reads. Around 10-40M total reads were obtained/ChIPseq sample whereof around 88-94% were uniquely mapped.
Antibodies	Anti-ATF1 C (cat#LS-C351857, LS Bio, lot#166109), -ATF1 N (cat#A303-034A, Bethyl laboratories, lot#2), -EWSR1 (C-9, cat#sc-48404, Santa Cruz, lot# J2517), -TFAP2A (3B5, cat#sc-12726, Santa Cruz, lot#H1220), -SOX10 (cat#PA5-40697, Invitrogen, lot#WH3347801) , -MITF (cat#91201, Active motif, Lot#30918002), -H3K4me3 (cat#07-473, Millipore, lot#2648189), -H3K27ac (cat#39133, Active motif, lot#31814008), -H3K4me1 (cat#ab8895, Abcam. lot#GR159018-1), -p300 (D2X6N, cat#54062, Cell signaling) and -V5 (D3H8Q, cat#13202, Cell signaling, lot#5).
Peak calling parameters	For ChIPseq, peaks were first called with MACS2 version 2.2.6 with an FDR of 1e-7 for ATF1 C, TFAP2A, SOX10, MITF, ATF1 N, V5 and H3K4me3, and 1e-4 for EWSR1, and the corresponding input samples. MACS peaks were then subjected to a second round of selection using the method described in Methods.<br>For HiChIPseq, the hichipper tool v 0.7.7 was used for loop calling by H3K27ac ChIP-seq peaks (q-value <= 0.00001), applying the parameters; max distance 100000000, read length 100.
Data quality	FastQC was used to evaluate data quality. For DTC1 and SU-CCS-1 wt ChIP-seq, only peaks present in both cell lines and both replicates were used for subsequent analyses.
Software	ChIPseq: TrimGalore v 0.6.4, STAR v 2.5.0a, MACS2 v 2.2.6, DeepTools v 3.5.0, HOMER v 4.11.1, bedtools v 2.27.1, R v 4.1.0.<br>HiChIPseq: hichipper v 0.7.7, Bioconductor v 3.13 DESeq2 and Diff-Loop tools.
